# Pineal Photoreceptor Cells Are Required for Maintaining the Circadian Rhythms of Behavioral Visual Sensitivity in Zebrafish

**DOI:** 10.1371/journal.pone.0040508

**Published:** 2012-07-16

**Authors:** Xinle Li, Jake Montgomery, Wesley Cheng, Jung Hyun Noh, David R. Hyde, Lei Li

**Affiliations:** 1 Department of Biological Sciences, Center for Zebrafish Research, University of Notre Dame, Notre Dame, Indiana, United States of America; 2 Tianjin Key Laboratory of Animal Models and Degenerative Neurological Diseases, Nankai University, Tianjin, China; Pennsylvania State University, United States of America

## Abstract

In non-mammalian vertebrates, the pineal gland functions as the central pacemaker that regulates the circadian rhythms of animal behavior and physiology. We generated a transgenic zebrafish line [Tg(Gnat2:gal4-VP16/UAS:nfsB-mCherry)] in which the *E. coli* nitroreductase is expressed in pineal photoreceptor cells. In developing embryos and young adults, the transgene is expressed in both retinal and pineal photoreceptor cells. During aging, the expression of the transgene in retinal photoreceptor cells gradually diminishes. By 8 months of age, the Gnat2 promoter-driven nitroreductase is no longer expressed in retinal photoreceptor cells, but its expression in pineal photoreceptor cells persists. This provides a tool for selective ablation of pineal photoreceptor cells, i.e., by treatments with metronidazole. In the absence of pineal photoreceptor cells, the behavioral visual sensitivity of the fish remains unchanged; however, the circadian rhythms of rod and cone sensitivity are diminished. Brief light exposures restore the circadian rhythms of behavioral visual sensitivity. Together, the data suggest that retinal photoreceptor cells respond to environmental cues and are capable of entraining the circadian rhythms of visual sensitivity; however, they are insufficient for maintaining the rhythms. Cellular signals from the pineal photoreceptor cells may be required for maintaining the circadian rhythms of visual sensitivity.

## Introduction

The pineal gland, which contains light-sensitive photoreceptor cells, plays important roles in the regulation of circadian rhythms in animal behavior and physiology [1], [2]. In zebrafish, the pineal gland develops during early embryonic stages [3]. The pineal gland is located in the dorsal diencephalon, and it contains both rod and cone photoreceptor cells [4–6]. The pineal photoreceptor cells share great homologies with retinal photoreceptor cells, such as cell morphology, the expression of opsin proteins, and responses to light stimuli [7, 8]. The pineal photoreceptor cells function autonomously with strong rhythmic patterns. For example, in cultured zebrafish pineal glands held in constant darkness, robust day-night rhythms in melatonin release and the expression of serotonin-n-acetyl-transferase are observed, suggesting the involvement of endogenous circadian clocks in pineal photoreceptor cells [9], [10].

In zebrafish, the behavioral visual sensitivity fluctuates between the day and night. Under a normal light-dark (LD) cycle, the zebrafish are most sensitive to light in the afternoon and least sensitive at night and in the early morning [11]. This pattern of visual threshold fluctuation persists in fish kept in the constant dark (DD) or constant light (LL), suggesting that the behavioral visual sensitivity is regulated by endogenous circadian clocks [11], [12]. The expression of photoreceptor cell-specific opsin mRNA also shows robust circadian rhythms, which correlate to the fluctuation patterns of behavioral visual sensitivity [13]. It has been demonstrated that the circadian oscillators that regulate photoreceptor cell functions (such as the expression of rhodopsin promoters) reside in the photoreceptor cells [14], [15]. Interestingly, the oscillations of individual photoreceptor cells do not synchronize. In a 24-hour period of DD, the expression of rhodopsin in individual photoreceptor cells may peak in the late afternoon or early morning. Alterations of cellular signals (e.g., activation of dopamine receptors or decrease in Ca^2+^ influx) synchronize the circadian oscillation among different photoreceptor cells [14]. This suggests that although photoreceptor cells contain circadian oscillators, signals from other sources (e.g., central pacemaker) may be required for maintaining the overall circadian rhythms of visual sensitivity.

The circadian nature of pineal photoreceptor cells, the day-night fluctuation of opsin expression and the oscillation of retinal sensitivity have been well documented. However, the fundamental mechanisms that regulate the circadian rhythms of visual sensitivity and the possible involvement of pineal central pacemakers in the regulation of the circadian rhythms of visual system functions remain to be examined. We generated a transgenic zebrafish line in which the pineal photoreceptor cells can be selectively ablated, thereby allowing functional studies of the effects of pineal photoreceptor cell ablation on the circadian rhythms of visual sensitivity. Taking advantage of the *E. coli* nitroreductase/metronidazole cell ablation system, we generated transgenic fish that express nitroreductase under the transcriptional control of cone photoreceptor cell-specific promoter Gnat2 [16], [17]. In developing embryos and young adults, the transgene is expressed in both retinal and pineal photoreceptor cells. After 8 months of age the expression of the transgene in the retina is no longer evident, but its expression in pineal photoreceptor cells persists. This provides a tool for selective ablation of pineal photoreceptor cells by treating the transgenic fish with metronidazole, i.e., pineal photoreceptor cells are destroyed due to the metronidazole-mediated conversion of nitroimidazole into cytotoxic agents [18]–[25]. In the current study, we investigated the effects of pineal photoreceptor cell ablation on the circadian rhythms of behavioral visual sensitivity in zebrafish. We demonstrated that retinal photoreceptor cells are sufficient to entrain the circadian rhythms of behavioral visual sensitivity; however, cellular signals from pineal photoreceptor cells are required for maintaining the rhythms.

**Figure 1:**
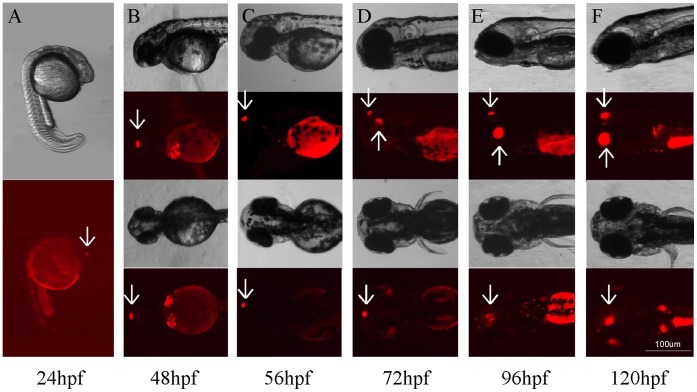
Bright-field and fluorescent images of developing embryos at different developmental stages that show Tg(Gnat2:gal4-VP16/UAS:nfsB-mCherry) transgene expression in the pineal gland and retina. (**A**) At 24 hpf, mCherry was detected in the pineal gland (arrow). (**B**) Strong mCherry expression was seen in the pineal gland (arrows). (**C–F**) Between 56 and 120 hpf, mCherry was detected in both the pineal gland (downward arrows) and retina (upward arrows). The intensity of transgene expression increased during embryonic development.

**Figure 2:**
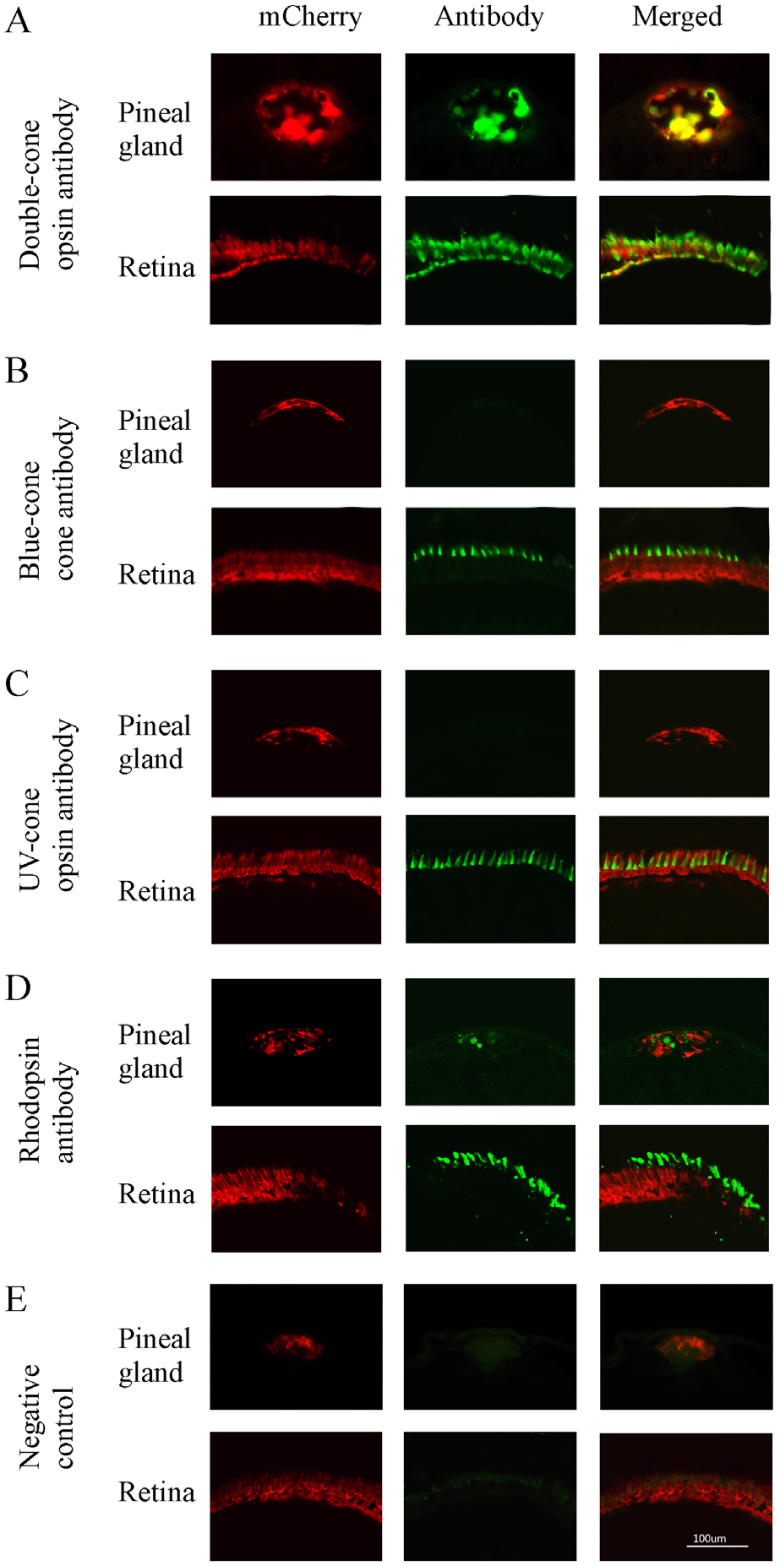
Cryostat sections of pineal gland and retina of transgenic zebrafish (2-month old) that show mCherry (left panels), opsin antibody immunoreactivity (middle panels), and co-localization of mCherry and opsin antibodies (right panels). (**A**) mCherry and double-cone opsin immunoreactivity in the pineal gland and retina. mCherry and double-cone opsin antibodies were co-localized in the pineal and retinal photoreceptor cells. (**B, C**) mCherry, blue- and UV-cone opsin immunoreactivity in the pineal gland and retina. Blue-cone and UV-cone antibody immunoreactivity were not seen in the pineal gland, but were detected in the retina where they co-localized with mCherry. (**D**) mCherry and rhodopsin immunoreactivity in the pineal gland and retina. Rhodopsin antibody immunoreactivity was detected in both pineal and retinal rod photoreceptor cells, but did not co-localize with mCherry. (**E**) Fluorescent images of pineal and retinal sections that were processed without primary antibodies (negative controls).

**Figure 3:**
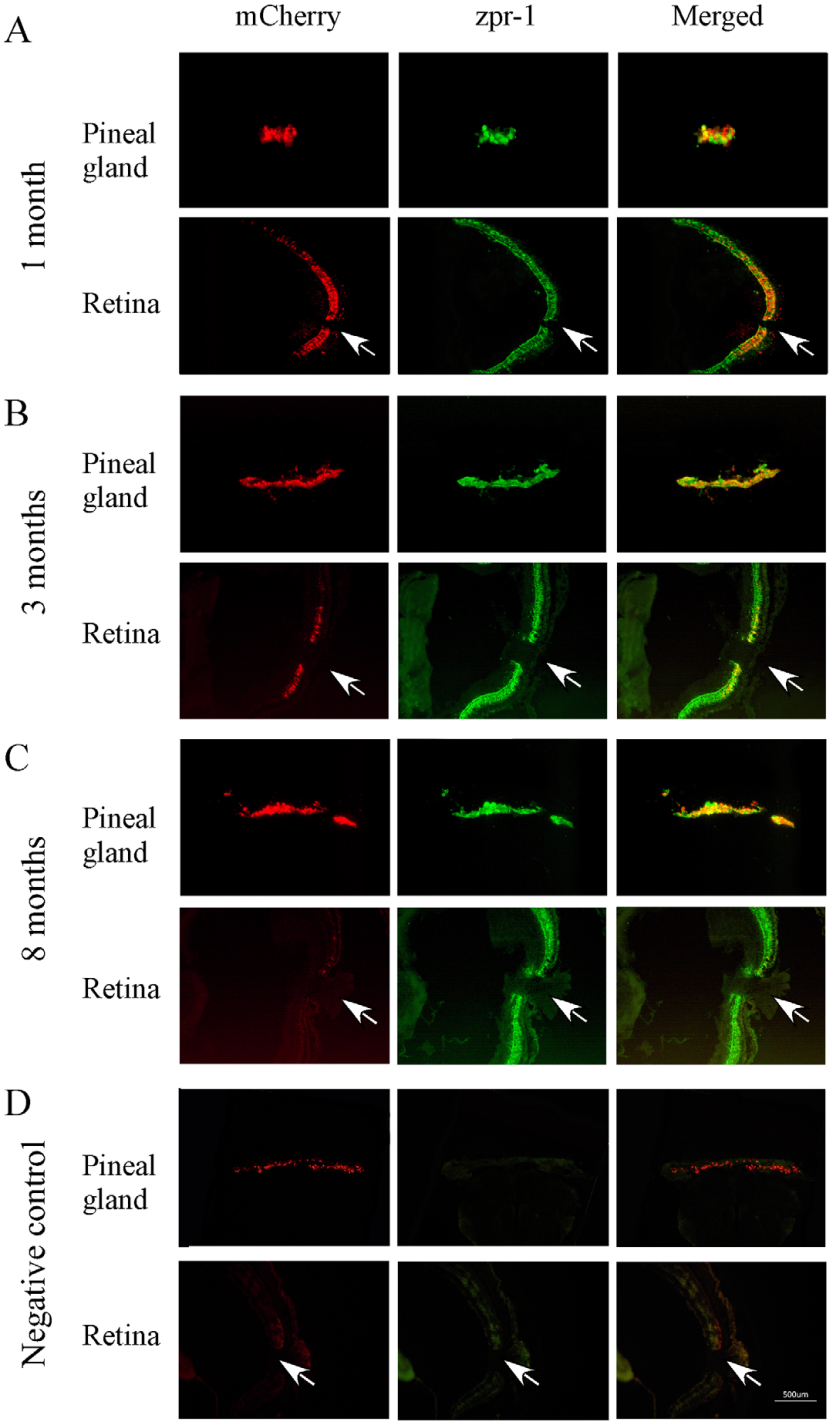
Cryostat sections of pineal gland and retina of transgenic zebrafish (1, 3 and 8 months) that show mCherry (left panels), double-cone opsin antibody (zpr-1) immunoreactivity (middle panels), and co-localization of mCherry and double-cone opsin antibody (right panels). (**A**) At 1 month of age, strong transgene expression was seen in the pineal gland and retina. mCherry and zpr-1 immunoreactivity were co-localized (**B**) At 3 months, the expression of mCherry decreased in the retina, but increased in the pineal gland. Co-localized of mCherry and zpr-1 immunoreactivity was detected in the pineal gland and central retina. (**C**) By 8 months, mCherry was detected in the pineal gland, but not in the retina. (**D**) Fluorescent images of pineal and retinal sections (from 8-months-old animals) that were processed without primary antibodies (negative controls). Arrows point the optic nerve.

## Results

### The Transgene is Expressed in Retinal and Pineal Photoreceptor Cells in Developing Embryos

We generated transgenic zebrafish Tg (Gnat2:gal4-VP16/UAS:nfsB-mCherry) that express *E. coli* nitroreductase and mCherry in cone photoreceptor cells. In developing embryos, the first transgene expression (reported by mCherry) was detected in the pineal gland at approximately 22–24 hours post-fertilization (hpf) (Fig. 1A). By 48 hpf, mCherry was detected in the retina, at which time retinal cone photoreceptor cells were developed (Fig. 1B). The intensity of transgene expression in the pineal gland and retina increased during embryonic stages (between 56 and 120 hpf; Fig. 1C–F). We also observed non-specific auto-fluorescence in the yolk and cardiovascular areas (Fig. S1).

We examined the cell types that express the transgene in the pineal gland and retina in young adult animals (2 months old) by co-localization of mCherry with various opsin antibodies. In the pineal gland, we observed strong immunoreactivity of zpr-1 antibody (which labels double-cone photoreceptor cells) (Fig. 2A). The expression of mCherry co-localized with zpr-1, suggesting that the transgene is expressed in double-cone-like photoreceptor cells. We did not detect blue-cone or UV-cone opsin expression in the pineal gland (Fig. 2B, C). Weak rhodopsin expression was detected, but it did not co-localize with mCherry (Fig. 2D). In the retina, mCherry was detected in all the cone cell types, and it co-localized with different cone opsin antibodies (zpr-1, blue- and UV-cone opsin antibodies, respectively) (Fig. 2A–C). We did not observe co-localization of mCherry and rhodopsin antibodies (Fig. 2D).

### The Expression of the Transgene is Diminished in Retinal Photoreceptor Cells in Adult Animals

We examined transgene expression in zebrafish at different ages. In developing larvae and young adults (1 month old), the transgene was expressed in cone photoreceptor cells in the pineal gland and retina. In the pineal gland and retina, co-localization of mCherry and zpr-1 antibodies was observed (Fig. 3A).

**Figure 4:**
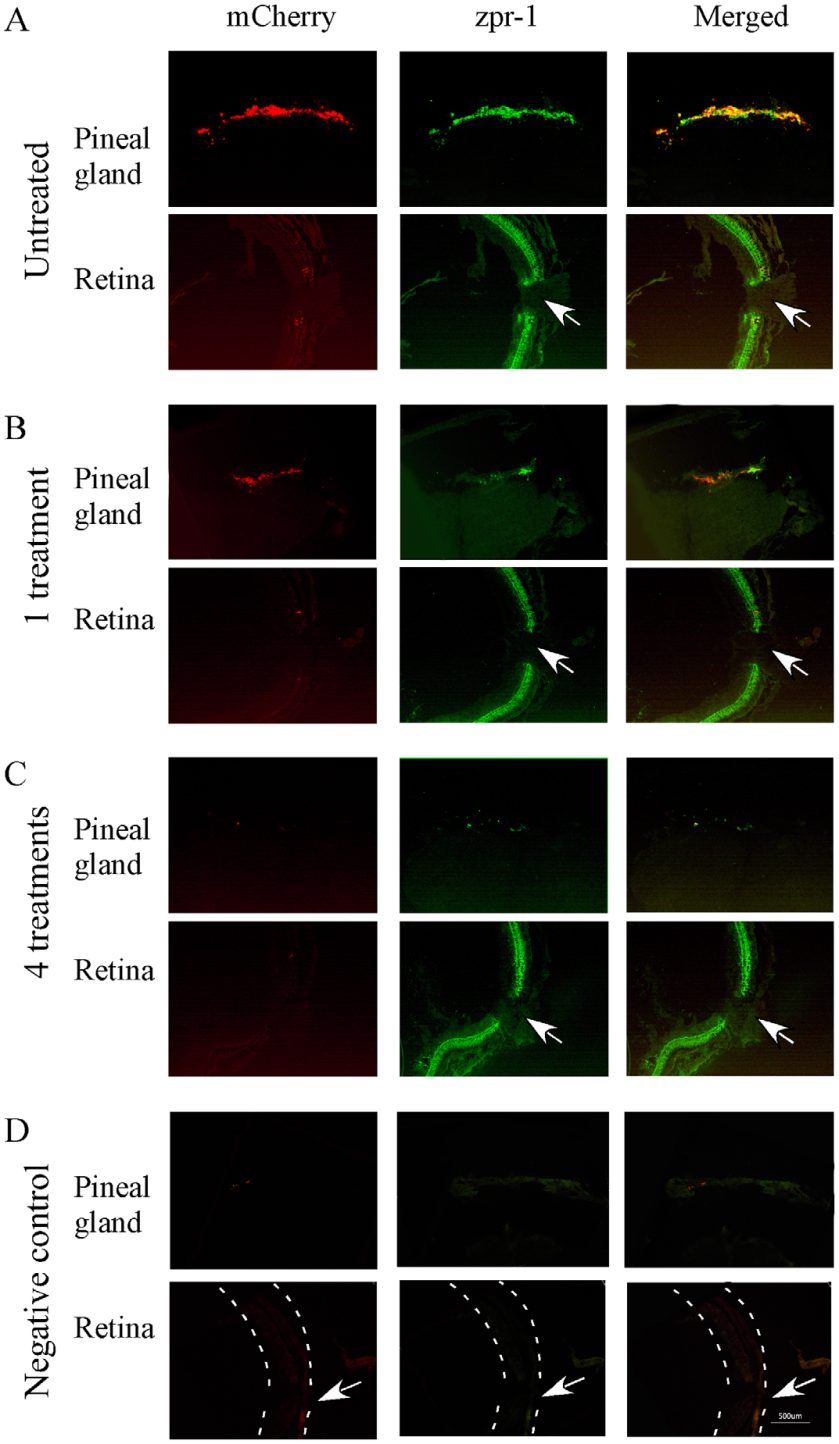
Cryostat sections of pineal gland and retina of transgenic zebrafish (8-month old) that show mCherry (left panels), double-cone opsin antibody immunoreactivity (zpr-1, middle panels), and co-localization of mCherry and opsin antibody (right panels) before and after the treatment with metronidazole. (**A**) Pineal and retinal sections from untreated transgenic fish. Strong mCherry expression was detected in the pineal gland, but not in the retina. zpr-1 immunoreactivity was seen in both pineal and retinal photoreceptor cells. (**B**) After 1 metronidazole treatment, mCherry expression and zpr-1 immunoreactivity in the pineal gland were decreased. In the retina, strong zpr-1 immunoreactivity was detected. (**C**) After 4 metronidazole treatments, the expression of mCherry and zpr-1 immunoreactivity in the pineal gland was completely diminished. Strong zpr-1 immunoreactivity was detected in the retina. (**D**) Fluorescent images of pineal and retinal sections that were processed without primary antibodies (negative controls). Dashed lines outline the retina. Arrows point to the optic nerve.

**Figure 5:**
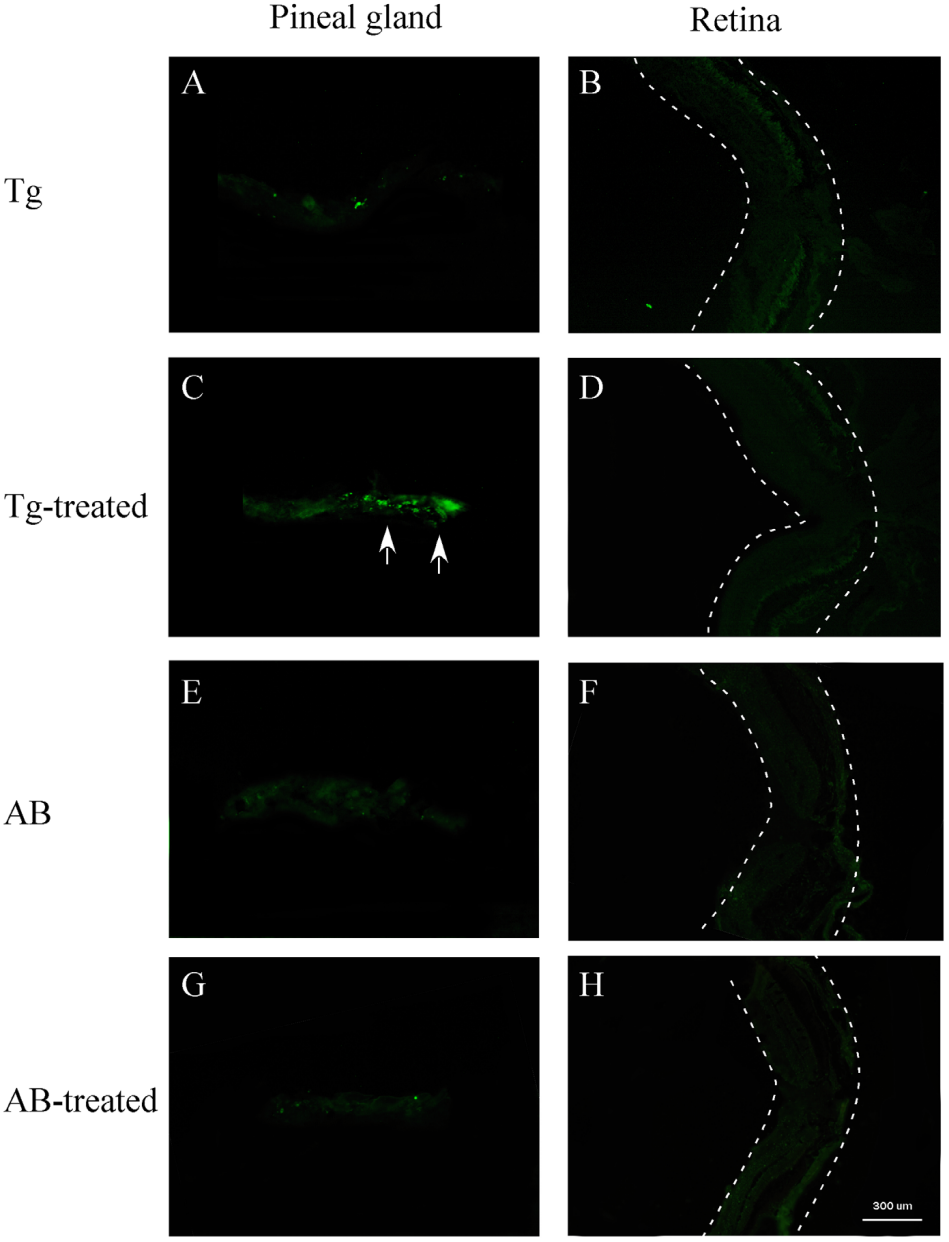
TUNEL labeling of pineal and retinal sections of metronidazole-treated and untreated transgenic (Tg) and wild-type (AB) zebrafish (8 months old). (**A, B**) Pineal and retinal sections of untreated transgenic fish. No obvious cell death was detected. (**C, D**) Pineal and retinal sections of transgenic zebrafish after 4 metronidazole treatments. In the pineal gland, TUNEL-positive cells were detected (arrows). No cell death was seen in the retina. (**E–H**) Pineal and retinal sections from untreated and metronidazole-treated wild-type zebrafish. No cell death was seen in the pineal gland or retina. Dashed lines outline the retina.

**Figure 6:**
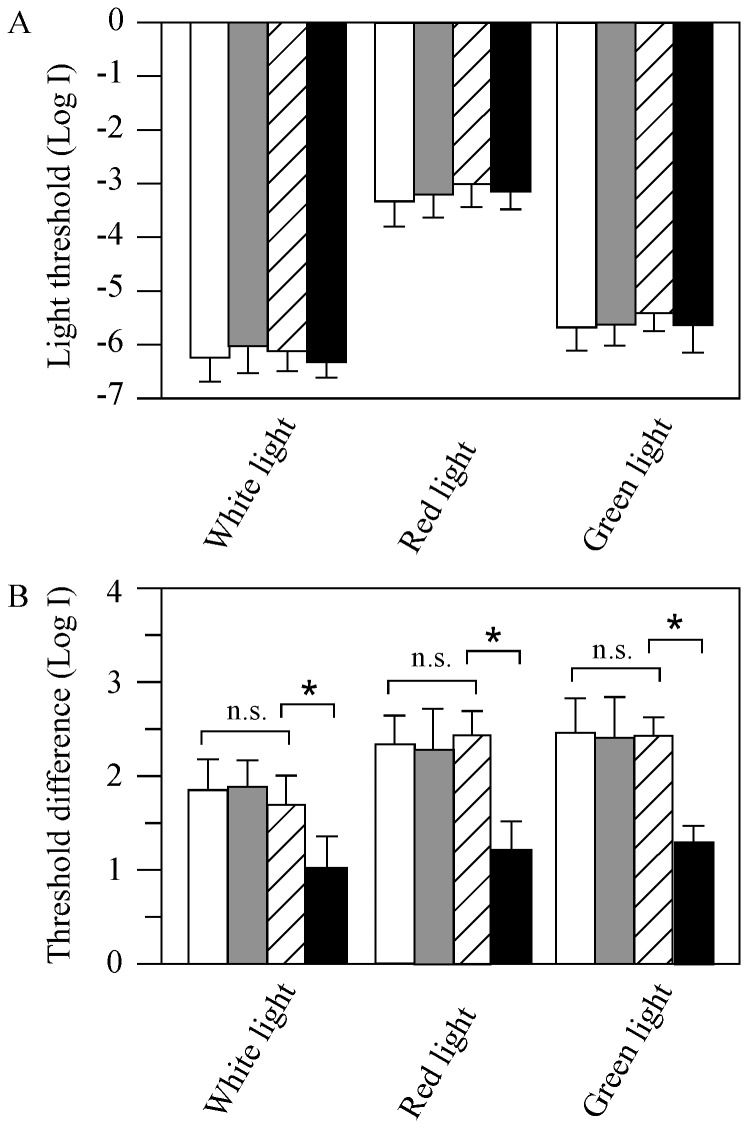
Behavioral visual thresholds of wild-type (white bars), metronidazole-treated wild-type (gray bars), transgenic (hatched bars) and metronidazole-treated transgenic fish (black bars) measured under white, red and green illumination, respectively. (**A**) Absolute visual thresholds measured at 6pm in white, red and green light in animals kept in the normal LD. Under either light conditions, no statistic differences in thresholds were seen between wild-type, metronidazole-treated wild-type, transgenic and metronidazole-treated transgenic animals. (**B**) Threshold difference between 3am and 7pm measured in wild-type, metronidazole-treated wild-type, transgenic and metronidazole-treated transgenic fish, respectively. The difference in visual threshold between 3am and 7pm decreased in metronidazole-treated transgenic fish tested under either light conditions. Data represent the Means ± SE (n = 12). * p<0.05; n.s., no statistic difference.

In zebrafish older than 3 months, the expression of the transgene in the retina was reduced in comparison to the expression in young adult animals. Often, the expression of the transgene was restricted to the central retina, near the optic nerve. Co-localization of mCherry and zpr-1 immunoreactivity was detected in the pineal gland and central retina (Fig. 3B). By 8 months, the expression of the transgene was completely diminished in the retina, i.e., no mCherry expression was detected, whereas its expression in the pineal gland remained unchanged (Fig. 3C). During this time period (between 3 and 8 months of age), we did not observe cell death in the retina, indicating that the loss of mCherry was due to diminished transgene expression, rather than cone photoreceptor cell apoptosis.

### Ablation of Pineal Photoreceptor Cells Does Not Affect Visual Sensitivity

In adult zebrafish older than 8 months, the transgene is only expressed in the pineal gland. This provides a tool for selective ablation of pineal photoreceptor cells without affecting retinal photoreceptor cells. We ablated pineal photoreceptor cells by treating the transgenic fish (>8 months) with metronidazole. After 4 metronidazole treatments, the expression of the mCherry in the pineal gland was diminished (Fig. 4A–C). We observed cell death (by TUNEL labeling) in the pineal gland. We did not obverse cell death in the pineal gland or retina in untreated transgenic fish or wild-type animals (Fig. 5). Cell loss was seen in the pineal gland in metronidazole-treated transgenic animals (by DAPI labeling, Fig. S2).

**Figure 7:**
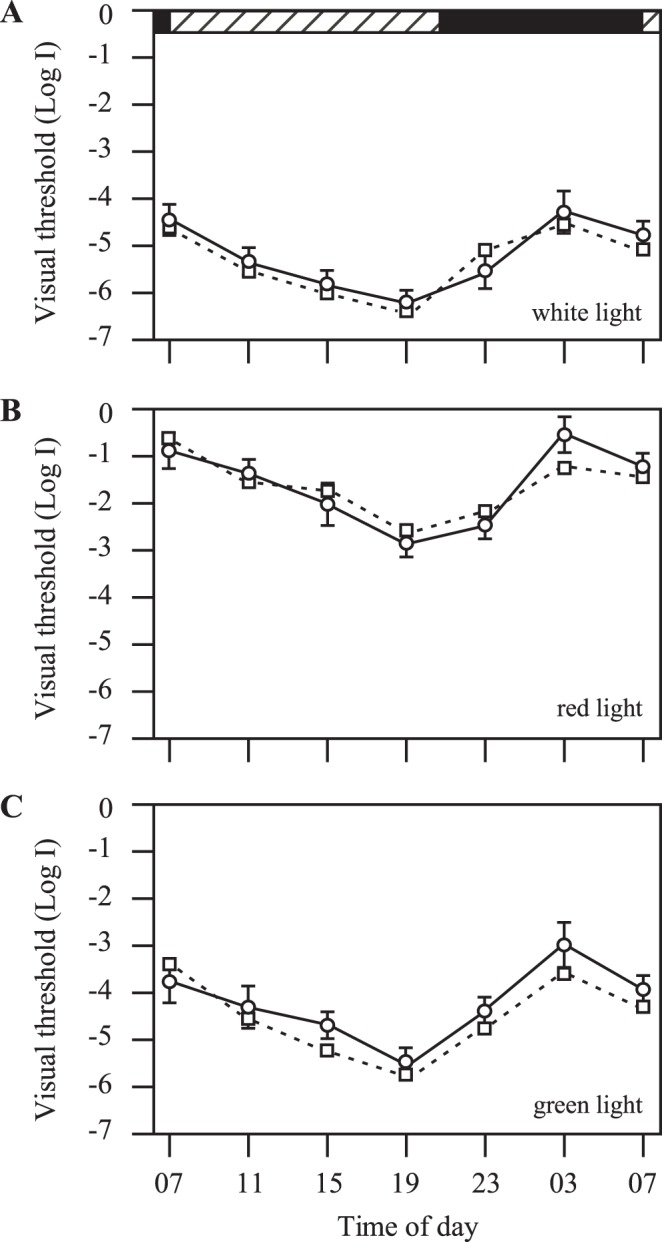
Circadian rhythms of behavioral visual sensitivity in transgenic fish (circles, n = 12) and metronidazole-treated transgenic zebrafish after 25-min of light exposures in the early morning (squares; n = 12). Threshold measurements were performed with white (**A**), red (**B**) and green light (**C**), respectively. Horizontal hatched and black bars on the top of the panel represent subjective day and night, respectively. Data represents the Means ± SE.

**Figure 8:**
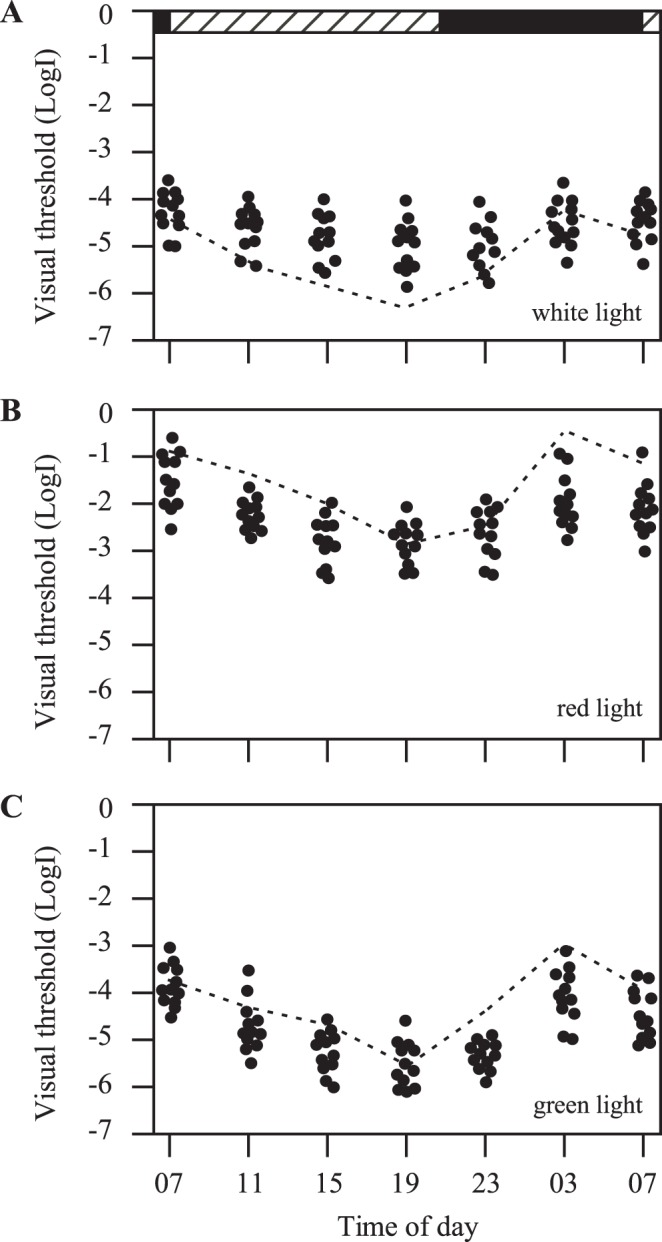
Behavioral visual thresholds measured in white, red and green light, respectively, in transgenic zebrafish after metronidazole treatments. Each circle represents a visual threshold measurement from one fish (n = 12). When measured with white light, visual thresholds increased in most times of the day and night, except at 3 and 7am, at which times the thresholds were already at the highest levels (**A**). Under red and green light illumination conditions, visual threshold decreased in the day and night, except 7pm, at which time the thresholds were already at the lowest levels (**B, C**). Dashed lines represent visual thresholds obtained from untreated control fish. Horizontal hatched and black bars on the top of the panel represent subjective day and night, respectively.

**Figure 9:**
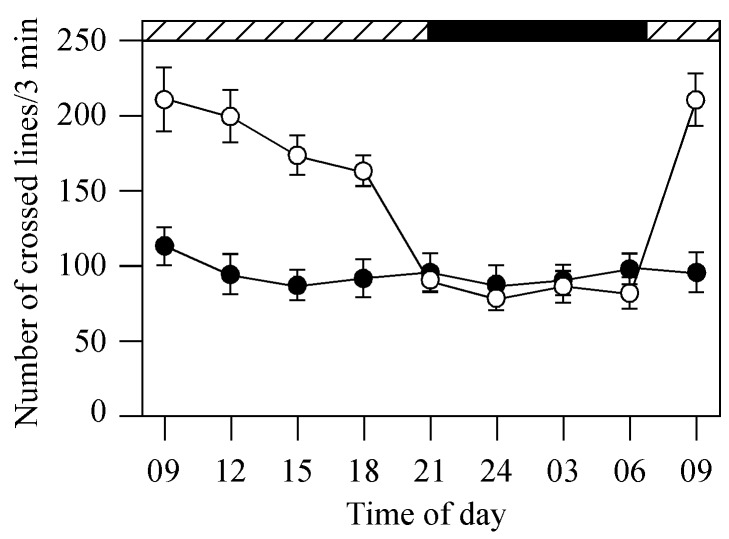
Locomotor activities of control (untreated transgenic fish; white circles, n = 12) and metronidazole-treated transgenic fish (black circles, n = 12) at different times of the day and night in DD. In the control group, the fish were more active in the day than in the night. In metronidazole-treated fish, swimming activities were similar in the day and night. Horizontal hatched and black bars on the top of the panel represent subjective day and night, respectively. Data represents the Means ± SE.

Depletion of pineal photoreceptor cells did not affect zebrafish visual sensitivities. When measured at 6 pm (in the normal LD, when the fish are most sensitive to light stimuli) [11], the absolute behavioral visual thresholds of wild-type fish (AB strain), metronidazole-treated AB fish, untreated- and metronidazole-treated transgenic fish were –6.2±0.3, –6.0±0.3, –6.1±0.3, and −6.2±0.2 log units, respectively (Fig. 6A). This suggests that the absolute rod sensitivity was not affected after pineal photoreceptor cell ablation. The behavioral cone sensitivities measured using narrow-band color filters were also similar in wild-type AB fish, metronidazole-treated AB fish, untreated and drug-treated transgenic zebrafish. When examined using a 625-nm wavelength cut-off filter, for example, the visual thresholds of wild-type AB fish, metronidazole-treated AB fish, untreated- and metronidazole-treated transgenic fish were −3.2±0.2, −3.1±0.2, −3.0±0.3, and −3.1±0.2 log units, respectively. When examined using a 500-nm wavelength cut-off filter, the behavioral visual thresholds of wild-type AB fish, metronidazole-treated AB fish, untreated- and metronidazole-treated transgenic fish were −5.7±0.3, −5.7±0.3, −5.4±0.2, and −5.6±0.3 log units, respectively (Fig. 6A).

### Ablation of Pineal Photoreceptor Cells Disrupted the Circadian Rhythms of Visual Sensitivity

In wild-type zebrafish, the behavioral visual sensitivity fluctuates between the day and night, i.e., the fish are most sensitive to light in the afternoon and least sensitive at night and in the early morning. From 6pm to 3am, the behavioral visual threshold increases approximately 2.0 log units [11]. This pattern of visual threshold fluctuation persists in animals kept in DD and LL conditions, suggesting the involvement of endogenous circadian clocks (see also Fig. S3). We measured visual sensitivity of Tg (Gnat2:gal4-VP16/UAS:nfsB-mCherry) fish at different times in the day and night while the fish were kept in DD. Similar to wild-type zebrafish, in the transgenic fish the visual sensitivity fluctuated between the subjective day and night (Fig. 7A). In DD, the fish were least sensitive to light at subjective night and early morning. During the day, visual sensitivity levels gradually increased. By 6pm, visual sensitivity increased to levels approximately 1.5–2.0 log units above the sensitivity levels measured in the early morning hours (3am), similar as seen in wild-type fish. In the evening, the visual sensitivity levels began to decrease. It continually decreased at night, and reached the least sensitive level at 3am (Fig. 7A). Similar patterns of visual threshold fluctuations were observed when measured using the narrow-band 625 nm and 500 nm wavelength filters. In both cases, the transgenic fish were most sensitive in the subjective late afternoon (7pm) and least sensitive in the early morning (3am and 7am) (Fig. 7B, C). Between these two time points, the difference in behavioral visual threshold was 1.7±0.4, 2.4±0.3, 2.4±0.3 log units, respectively, when tested under white, red and green light, similar as seen in wild-type animals (Fig. 6B).

In the absence of pineal photoreceptor cells (after metronidazole treatments), the circadian rhythms of behavioral visual sensitivity were dampened out. When tested using white light (n = 12), for example, the behavioral visual thresholds increased at most time points, except at 3am and 7am when the visual thresholds were already at the highest levels (Fig. 8A). Between the day and night, the difference in visual threshold in metronidazole-treated fish was reduced to approximately 0.9±0.3 log unit (Fig. 6B). Metronidazole-treatments produced no effects in the circadian rhythms of behavioral visual sensitivity in untreated transgenic or wild-type animals (Fig. 6B; S3).

The circadian rhythms of retinal cone sensitivity (measured using 625 nm and 500 nm wavelength narrow-band filters, respectively) were also dampened out after metronidazole treatments (Fig. 8B, C). This was largely due to visual threshold decreases (except in the late afternoon, when the fish were already most sensitive to light). When examined using red light, for example, at 3am all the tested fish (n = 12) showed decreased visual threshold levels when compared to the visual thresholds measured in untreated transgenic fish (Fig. 8B). When examined using green light, at 11pm and 3am all the tested fish (n = 12) showed decreased visual threshold levels (Fig. 8C). Between the subjective day and night, in metronidazole-treated fish the difference in behavioral visual thresholds were decreased to 1.2±0.3 and 1.3±0.2 log units, respectively, when measured using 625 nm and 500 nm wavelength filters (Fig. 6B). Metronidazole-treatments produced no effects in the circadian rhythms in cone sensitivity in untreated transgenic and wild-type animals (Fig. 6B; S3).

### Brief Light Exposures Restore the Circadian Rhythms of Behavioral Visual Sensitivity in Metronidazole-Treated Transgenic Zebrafish

We investigated whether light exposures could restore the circadian rhythms of visual sensitivity in metronidazole-treated transgenic zebrafish. In these experiments, the fish received 25-min light exposures at 7am (room fluorescent light). Thereafter, the fish were kept in DD for 24 hours. At 7am on the following day, the fish were tested for behavioral rod and cone sensitivity using white light, red (625 nm) and green (500 nm) light, respectively. The measurements were repeated in 4-hour intervals, and completed at 7am on the following day.

In 8 of 12 fish tested (with white light), the circadian rhythms of behavioral visual sensitivity were restored. During a 24-hour period of subjective day and night, the fish were most sensitive to light in the subjective afternoon, and least sensitive at subjective night and early morning, similar to the patterns of visual threshold fluctuation in wild-type and untreated transgenic control animals (Fig. 7A). In the remaining fish (n = 4), two fish showed no threshold fluctuations between the day and night, and two fish showed abnormal fluctuation patterns (i.e., they were most sensitive to light at night or in the early morning). After the short light exposures, the metronidazole-treated transgenic zebrafish also showed restored circadian rhythms of cone sensitivity when tested with red and green light (9 out of 12 fish in red light, and 8 out of 12 fish in green light). They were sensitive to light stimuli in the afternoon and not sensitive at night and in the early morning (Fig. 7B, C). The remaining fish showed no visual threshold fluctuation or shifted patterns of fluctuation.

### Ablation of Pineal Photoreceptor Cells Interrupts the Circadian Rhythms of Locomotor Behaviors

We evaluated the effects of pineal photoreceptor cell ablation on circadian rhythms of other activities in the transgenic fish, such as locomotion. Locomotor behaviors were assessed by monitoring the swimming activity of the fish. The fish were constrained in a 2-liter container (L×W×H: 30×10×5.5 cm) filled with 0.5 liter system water (see also [26]). Five vertical lines were drawn on the wall of the container at an equal distance. In a 3-min period, we counted the number of lines the zebrafish crossed. The experiments were conducted at different times in the day and night while the fish were kept in DD. In control groups (untreated transgenic fish), the fish was most active in the day and least active during the night (Fig. 9). At 9am, for example, swimming activities of the fish were approximately 3-fold higher than the swimming activities measured at midnight. In metronidazole-treated transgenic fish, however, swimming activities were decreased during the day and night. We did not observe fluctuations in locomotor behaviors between the day and night (Fig. 9).

## Discussion

In this study, we provide evidence that the pineal photoreceptor cells play important roles in maintaining the circadian rhythms of behavioral visual sensitivity in zebrafish. Taking advantage of the diminished transgene expression in retinal photoreceptor cells, we were able to selectively destroy pineal photoreceptor cells in the transgenic Tg(Gnat2:gal4-VP16/UAS:nfsB-mCherry) fish by metronidazole treatments. This provided a tool for functional studies of the potential roles of pineal photoreceptor cells in retinal sensitivity. In the absence of pineal photoreceptor cells, the behavioral visual sensitivity remained unchanged. However, when tested in the DD conditions, the circadian rhythms of behavioral rod and cone sensitivity were diminished. Brief light exposure applied in the early morning restored the circadian rhythms of behavioral visual sensitivity, suggesting that retinal photoreceptor cells are sufficient for entraining the circadian rhythms of visual sensitivity. However, cellular signals from pineal photoreceptor cells may be required for maintaining the circadian rhythms of visual sensitivity.

In the transgenic Tg(Gnat2:gal4-VP16/UAS:nfsB-mCherry) fish, we did not detect immunoreactivity of long-single (blue) cone or short-single (UV) cone opsin antibodies in the pineal gland. We observed weak rhodopsin immunoreactivity, suggesting that rod photoreceptor cells are present in the pineal gland. Because the rod photoreceptor cells did not express the transgene, metronidazole treatments did not affect the survival of rod photoreceptor cells (see also [22], [25]). However, we do not rule out the possibility that the light-induced restoration of the circadian rhythms in metronidazole-treated transgenic fish may partially be due to cellular signals produced from the rod photoreceptor cells in the pineal gland. For example, after light exposures the synthesis and release of melatonin from pineal rod photoreceptor cells may increase, thereby resetting the circadian rhythms of retinal sensitivity.

In metronidazole-treated transgenic fish, light-induced restoration of the circadian rhythms of visual sensitivity may also be due to increases in dopamine release from retinal dopaminergic interplexiform cells (DA-IPCs). Dopamine has long been known to play important roles in the regulation of retinal functions, including circadian visual sensitivity [27]. In a recent study, Yu et al [14] demonstrated that light exposures synchronize the circadian rhythms of rod photoreceptor promoter activity through dopamine-mediated Ca^2+^ signaling pathways. In cultured zebrafish retinas, brief light exposures increased dopamine release from DA-IPCs. Through dopamine D2 receptor-coupled cellular pathways, Ca^2+^ influx decreased in photoreceptor cells, leading to decreases in rhodopsin promoter activity in rod cells. Consequently, the circadian rhythms of photoreceptor cell sensitivity were synchronized.

We measured behavioral visual sensitivity in metronidazole-treated transgenic fish using different light sources. Red light (passed through a 626 nm wavelength cut-off filter) primarily activates red cone photoreceptor cells [28]–[30]. When using a 500 nm wavelength cut-off filter, however, both the rod and green cone photoreceptor cells may become activated (i.e., the maximum absorption of rod and green cone photoreceptor cells in zebrafish is 500 and 480 nm, respectively (see also [30]–[32]). Thus, the results from using green light filter may reflect the sensitivity of both rod and green cone cells. This notion is supported by the fact that the light thresholds measured with the 500 nm wavelength green light filter and white light are similar (−5.6 log units *vs* −6.2 log units). Normally, cone system sensitivities (i.e., when measured using the narrow-band 625 nm wavelength filter) are significantly lower than rod sensitivity (−3.2 log units *vs* −6.2 log units).

In DD conditions, at most of the times in the subjective day and night, the behavioral cone thresholds decreased in metronidazole-treated transgenic fish (except at 7pm, at which time the cone thresholds are already at the lowest level). This suggests that when the circadian clocks are functioning, they inhibit retinal cone sensitivity; when the circadian oscillators are ablated, cone sensitivity is increased. It has been demonstrated that in zebrafish kept in prolonged LL or DD, after the circadian rhythm of visual sensitivity is dampened out, the merged sensitivity level is close to the most sensitive levels measured in the late afternoon [11]. This suggests that functioning circadian clocks inhibit visual sensitivity. In a recent study, Emran et al [33] demonstrated that in zebrafish larvae, the cone sensitivity (measured by the electrophysiological ERG assay) is inhibited at night due to a circadian mechanism. The reduced cone sensitivity at night may be due to decreases in photoreceptor cell activity and disassembly of synaptic ribbons of cone pedicles.

Ablation of pineal photoreceptor cells affected not only the circadian rhythms of retinal sensitivity, but also the rhythms of other physiological activities, such as locomotor behaviors. This suggests that in zebrafish, the pineal photoreceptor cells function as a central pacemaker that regulates animal’s behavior and physiology. In addition to pineal photoreceptor cell signal pathways, other cellular signals in the retina may also be required in the regulation of the circadian rhythms of visual system function. The mechanisms that regulate the circadian rhythms of retinal rod sensitivity seemed different from those that regulate the rhythms of cone sensitivity. After metronidazole-treatment, at most time points in the day and night, the behavioral rod threshold increased (except at 3am, at which time the rod threshold is already at the highest level). It is not known why the ablation of pineal cone cells resulted in increases in rod threshold, rather than decreases as seen in retinal cone cells. It is possible that after the pineal cone photoreceptor cell ablation, cone-dominant light incremental sensitivity is impaired. As a result, the fish constantly remain in the “night” status, during which time the rod threshold remains high [11]. It is also possible that pineal signals that regulate retinal rod and cone sensitivities are separate. Metronidazole treatments destroyed the cone cells in the pineal gland, and interrupted the production of melatonin from pineal rod cells. As a consequence, pineal signals to the retina are impaired, and both rod and cone sensitivities are affected, but in different ways.

## Materials and Methods

### Fish Maintenance

Zebrafish (Danio rerio, wild-type AB and transgenic fish were maintained as previously described [34]. Normally, the fish were kept in cyclic light-dark (LD; room fluorescent light, from 07∶00 to 21∶00). For the DD experiments, the fish were removed from the LD at 21∶00 the day before the experiment and subsequently kept in the DD. All the experiments were performed according to the NIH guidelines for animals in research.

### Generation of Transgenic Zebrafish

The previously identified 3.2 kb zebrafish guanine nucleotide binding protein (G protein), alpha transducing activity polypeptide 2 (gnat2) promoter fragment was digested from a plasmid construct [17] with XhoI and BamHI. The restriction fragment was ligated into the XhoI and BamHI sites of a modified pT2KXIG vector [35] that contained a custom multiple cloning site and an SV40 late poly(A) sequence [36]. A 654 bp Gal4-VP16 fusion [37] was then ligated into the BamHI and NotI restriction sites between the promoter and poly(A) sequence. The resulting expression construct was purified with the QIAGEN Plasmid Maxi Kit (Qiagen, Valencia, CA), followed by phenol/chloroform (1∶1) extraction. Tol2 transposase mRNA was in vitro transcribed from the pCSTZ2.8 plasmid [35] using the SP6 mMessage mMachine kit (Ambion, Foster City, CA).

The pT2KXIG-gnat2:Gal4-VP16 expression construct and Tol2 transposase mRNA were resuspended in nuclease-free water at a combined concentration of 25 ng/µl each, and co-injected into 1–4 cell stage wild-type AB strain embryos. Injected fish were out-crossed to the Tg(UAS-E1b:nfsB-mCherry)jh17 line [37] and the progeny were screened for fluorescence to identify founders and to establish the Tg(gnat2:Gal4-VP16) nt21 line. Out-crosses of double-transgenic to wild-type fish produced fluorescence in 25% of the progeny, which is consistent with single insertions of each transgene. For the described research, the transgenic fish (embryos or adults) were obtained by selecting individuals that expressed mCherry at 3 dpf from crosses between the Tg(Gnat2:gal4-VP16/UAS:nfsB-mCherry) transgenic fish and wild-type AB zebrafish.

### Metronidazole Treatment

Metronidazole (Sigma-Aldrich, St. Louis, MO) was dissolved in regular tank water (Instant Ocean Salts in distilled water, 3 g/gal, pH = 7.2). The fish were treated at 28° in 1.0 liter tank water containing 10 mM metronidazole. After 24 hours of treatment, the fish were removed from the treatment tank and transferred into the recovery tank, which contained regular tank water. The fish were treated 4 times (in 2-day intervals) before the behavioral experiments were performed. This scheme of metronidazole treatment did not cause lethality in adult zebrafish. Complete ablation of the pineal photoreceptor cells after the metronidazole treatments were confirmed by examining the pineal gland (in whole-mount embryos or cryostat sections of adult brain tissues) with fluorescent microscopy (i.e., lack of mCherry) and TUNEL labeling (i.e., evidence of cell death).

### Immunolabeling

Procedures for immunolabeling were similar as previously described [38]. Embryos, adult eyes or brain tissues were fixed in 4% paraformaldehyde and embedded in OCT. Cryostat sections (adult retinas or pineal glands) were collected at 12 µm in thickness. The following primary antibodies were used: mouse monoclonal anti-double cone opsin (zpr-1, 1∶200); [University of Oregon], rabbit polyclonal anti-blue opsin (1∶250); rabbit polyclonal anti-UV opsin (1∶1000); rabbit polyclonal anti-rhodopsin (1∶5000) [38]. Secondary antibodies (Alexa Fluor 488, goat anti-mouse or rabbit) were used at a dilution of 1∶500. TUNEL labeling was performed using the ApoAlert DNA Fragmentation Assay Kit (Clontech, Mountain View, CA).

### Behavioral Visual Threshold Measurement

Methods for measuring zebrafish visual sensitivity were similar as previously described [39]. The test apparatus consisted of a circular transparent plastic container surrounded by a drum rotating at 10 rpm in either clockwise or counterclockwise directions. The inside of the drum was covered by white paper marked with a black segment. The drum was illuminated from above with white light, maximum intensity, log I = 425 µW/cm2. The intensity of the light was adjusted by changing neutral density filters in 0.5 log unit steps.

Normally, the fish swim slowly along the wall of the container. However, when encountered by the black segment rotating outside the container, the fish display robust escape responses, i.e., as soon as the black segment comes into view, the fish immediately turns and rapidly swims away. Usually, a judgment of whether the fish saw the black segment can be made in less than 10 seconds. To measure the behavioral visual sensitivity, we recorded the minimum light intensities that evoked behavioral escape responses. At least 5 escape responses in 8 encounters were required to record a threshold response. The measurement was performed under different light wavelengths: white light (rod sensitivity), red light (white light that passes a 625-nm wavelength narrow band cut-off filter; red cone sensitivity), green light (white light that passes a 500-nm wavelength narrow band cut-off filter; green cone sensitivity).

## Supporting Information

Figure S1 **The expression of auto-fluorescence in the yolk in a developing embryo.**
(EPS)

Figure S2 **DAPI staining of the pineal gland of metronidazole-treated and untreated adult fish.**
(EPS)

Figure S3 **Circadian rhythms of behavioral visual sensitivity of wild-type fish.**
(EPS)
